# Biodegradation and Removal of PAHs by *Bacillus velezensis* Isolated from Fermented Food

**DOI:** 10.4014/jmb.2104.04023

**Published:** 2021-05-21

**Authors:** Omme Fatema Sultana, Saebim Lee, Hoonhee Seo, Hafij Al Mahmud, Sukyung Kim, Ahyoung Seo, Mijung Kim, Ho-Yeon Song

**Affiliations:** 1 Department of Microbiology and Immunology, School of Medicine, Soonchunhyang University, Cheonan 31151, Republic of Korea; 2 Probiotics Microbiome Convergence Center, Soonchunhyang University, Asan 31538, Republic of Korea

**Keywords:** PAH degradation, PAH removal, *B. velezensis*, fermented food, probiotics, ring-hydroxylating dioxygenase

## Abstract

Polycyclic aromatic hydrocarbons (PAHs) are ubiquitous in the environment. They are highly toxigenic and carcinogenic. Probiotic bacteria isolated from fermented foods were tested to check their ability to degrade and/or detoxify PAHs. Five probiotic bacteria with distinct morphologies were isolated from a mixture of 26 fermented foods co-cultured with benzo(a)pyrene (BaP) containing Bushnell Haas minimal broth. Among them, *B. velezensis* (PMC10) significantly reduced the abundance of BaP in the broth. PMC10 completely degraded BaP presented at a lower concentration in broth culture. *B. velezensis* also showed a clear zone of degradation on a BaP-coated Bushnell Haas agar plate. Gene expression profiling showed significant increases of PAH ringhydroxylating dioxygenases and 4-hydroxybenzoate 3-monooxygenase genes in *B. velezensis* in response to BaP treatment. In addtion, both live and heat-killed *B. velezensis* removed BaP and naphthalene (Nap) from phosphate buffer solution. Live *B. velezensis* did not show any cytotoxicity to macrophage or human dermal fibroblast cells. Live-cell and cell-free supernatant of *B. velezensis* showed potential anti-inflammatory effects. Cell-free supernatant and extract of *B. velezensis* also showed free radical scavenging effects. These results highlight the prospective ability of *B. velezensis* to biodegrade and remove toxic PAHs from the human body and suggest that the biodegradation of BaP might be regulated by ring-hydroxylating dioxygenase-initiated metabolic pathway.

## Introduction

Polycyclic aromatic hydrocarbons (PAHs) are organic compounds made of two or more fused benzene rings [1]. PAHs are ubiquitous in the environment. They are usually formed by incomplete combustion of organic materials such as oil, coal, and gasoline. Due to their toxic, carcinogenic, and/or bioaccumulative nature, they can cause different diseases in the human body [2]. By changing or damaging genetic materials, PAHs can initiate the development of different cancers. Clinical studies have also shown PAHs' involvement in skin, stomach, liver, lung, bladder, and gastrointestinal cancers [3, 4]. Among different PAHs, benzo(a)pyrene, benz(a)anthracene, dibenzo(a,l)pyrene, benzo(b)fluoranthene, benzo(k)fluoranthene, chrysene, dibenz(a,h)anthracene, indeno(1,2,3-cd)pyrene, and naphthalene have been reported to be potent carcinogens by the Environmental Protection Agency (EPA) and other studies [4-7]. In addition, different mixtures comprising PAH compounds such as coal tar and vehicle exhaust have been reported by the International Agency for Research on Cancer as potential carcinogenic agents to humans [8]. Among them, benzo(a)pyrene and dibenz(a,h)anthracene have been reported to be the most carcinogenic [3]. Individuals can have both occupational and non-occupational exposure to PAHs through inhalation, ingestion, and dermal contact. A mixture of PAHs can also cause skin irritation and inflammation [9].

Probiotic bacteria are being used as beneficial agents for human health. They have been reported as a potential therapeutic option for degrading xenobiotics and removing toxic molecules [1, 10]. Lactic acid bacteria (LAB) from a Japanese fermented food have binding affinities for heterocyclic amines [11]. Traditionally, LAB are used in the fermentation process in the food industry as beneficial microorganisms. They are also used as therapeutic agents for specific human diseases [12]. Although human clinical trials remain limited, probiotic bacteria are currently attracting much attention as anti-genotoxic and anti-mutagenic functional food [13]. Several probiotics have also been reported to be able to neutralize chemical-induced genotoxic or mutagenic effects [14]. To identify a potential safe probiotic candidate that could degrade or remove PAHs from the human body or food, we co-incubated 26 Korean fermented foods with benzo(a)pyrene (BaP) for six successive 7-day incubations to find an effective probiotic that could degrade BaP. Among hundreds of PAHs, BaP is the best known and studied. It is frequently used as a PAH exposure marker [15]. Thus, we utilized BaP in the present study. Naphthalene is often used as a chemical model to study the PAH effect [16]. Thus, it was also selected in the present study. Isolated colonies were also subjected to GCMS analysis to evaluate BaP degradation efficacy. Additionally, expression levels of two enzymes, PAH ring- hydroxylating dioxygenases (PAH-RHDalpha) and 4-hydroxybenzoate 3-monooxygenase (HBMO), were analyzed by qRT-PCR. These enzymes are involved in the initial step of aerobic metabolism of PAH [17]. In addition, the PAH-binding ability of the best candidate from the degradation experiment was evaluated in aqueous conditions. Functional effects such as anti-inflammatory and antioxidative effects of the selected probiotic candidate, *B. velezensis*, were also evaluated.

## Materials and Methods

### Sample Selection

In this study, we collected a total of 26 fermented foods from different parts of South Korea. These fermented foods could be classified into four different categories: fermented sauce (*n* = 18), kimchi (*n* = 3), cheese (*n* = 3), and vinegar (*n* = 2). Samples were preserved at 4°C in the laboratory for further experiments.

### Enrichment and Isolation of PAH-Degrading Bacteria from Fermented Foods

Each gram of 26 fermented foods was taken separately into a tube, added with 26 ml of PBS, and mixed vigorously. Then, 5 ml of the mixed food solution was transferred to 45 ml Bushnell-Haas (BH) minimal medium containing 0.25% or 250 mg/l PAH (benzo(a)pyrene). The medium was supplemented with 5 ml of trace metal solution and 100 μl of vitamin solution per liter of media. The trace metal solution (1 L) in distilled water was prepared by mixing 200 mg of FeSO_4_•7H_2_0, 10 mg of ZnSO_4_•7H_2_0, 3 mg of MnCl_2_•4H_2_0, 20 mg of CoCl_2_•6H_2_0, 1 mg of CuCl_2_•2H_2_0, 2 mg of NiCl_2_•6H_2_0, 500 mg of Na_2_MoO_4_•2H_2_0, and 30 mg of H3BO3. A vitamin stock solution contained (per 100 ml of distilled water) 2 mg of biotin, 2 mg of folic acid, 5 mg of thiamine HCl, 5 mg of D-calcium pantothenate, 5 mg of vitamin B12, 5 mg of riboflavin, 20 mg of niacin, 3 mg of pyridoxal HCl, and 2 mg of paraaminobenzoic acid [18]. Then, 15 *g* bacto (BD Difco, USA) agar was added, and the mixture was autoclaved to prepare Bushnell Haas agar (BHA).

PAH-degrading bacteria were isolated with a successive culture method, as reported earlier [19]. Briefly, mixed food samples were cultured in BH broth containing 0.25% PAH as the sole carbon source and incubated at 37°C in a shaking incubator (100 rpm) for seven days. Next, 5.0 ml of phase 1 culture was transferred to another 45 ml fresh BH broth containing 0.25% PAH and incubated for another week under similar conditions. This culture was repeated for another five consecutive times maintaining similar conditions to enable the bacteria to withstand the imposed stress.

### Identification of PAH-Degrading Bacteria by 16S rRNA Sequencing

Bacteria that survived in the presence of 0.25% PAH as a sole source of carbon were spread onto a nutrient agar plate. Following incubation at 37°C, colonies with distinct morphologies were selected and stored at -80°C for further experiment.

Distinct colonies were identified by 16S rRNA gene sequencing. Genomic DNA was extracted and purified as previously described using a QIAamp DNA Mini Kit (Qiagen, Germany) following the manufacturer’s protocol [20]. Universal primers 27F (5’-GAGAGTTTGATCCTGGCTCAG-3’) and 1495R (5’-CTACGGCTACCTTGT TACGA-3’) were used for 16S rRNA gene amplification and sequencing (Eurofins MWG Operon, Germany). Sequences were compared with those contained in the Ribosomal Database Project. Isolates were identified according to the Clinical and Laboratory Standards Institute (CLSI) guidelines [21] with an identity score of 99%.

### Whole Genome Sequencing

Genomic DNA was extracted from PMC10 using a QIAamp DNA Mini Kit (Qiagen) following the manufacturer’s protocol. PacBio sequencing of the isolated genomic DNA was conducted at Chunlab Inc. (Korea). Obtained sequence data were assembled by PacBio SMRT Analysis 2.3.0 using HGAP2 protocol (Pacific Biosciences, USA). Finally, contigs obtained from the PacBio sequencing analysis were circularized using Circlator 1.2.0 (Sanger Institute, UK).

### Quantifying PAH Degradation by GCMS Analysis

Isolated bacteria were inoculated into LB broth and incubated at 37°C overnight. The enriched culture was centrifuged at 4,000 *g* for 10 min, and the pellet was re-suspended with 3 ml BH medium. Then 1,000 μl of bacterial culture was added to 29 ml BH medium containing PAHs (10 μg/ml). After 20 days of incubation at 37°C in a shaking incubator, the concentration of PAHs was determined by GCMS analysis. Briefly, n-hexane was used as an extraction solvent and injected into the aqueous sample with acetone. The ratio of sample-to-extraction solvent was 1:10. Following 2 min of extraction, to break the emulsion, a second aliquot of acetone was injected into the solution as a demulsifier. The emulsion rapidly became clear and separated into two phases. The organic upper phase was collected and analyzed using an Agilent GC-5975C GC–MS system equipped with a Shimadzu AOC-20i autosampler and a DB-5ms (J&W Scientific, USA) fused silica capillary column (30 m × 0.25 mm internal diameter (i.d.), 0.25 μm film thickness). Helium with a purity of 99.9999% was employed as the carrier gas at a flow rate of 1.0 ml/min. Next, 1 μl of each sample was injected in a splitless mode (10 ml/min, 1 min). The injector temperature and interface temperature were maintained at 280 and 300°C, respectively. Initially, the GC oven was held at 70°C for 2 min and then shifted to 190°C at 15°C/min. Following holding at 190°C for 1 min, the oven was programmed to 260°C at 10°C/min. Finally, it was programmed to 285°C at 5°C/min and held for 5 min. The solvent cut time was 6 min. Masses monitored by the detector were set as follows: (6–8) min, m/z (128, 129, 127, 102); (8–9.5) min, m/z (152, 153, 151, 154); (9.5–10.8) min, m/z (166, 165, 167, 139); (10.8–13) min, m/z (178, 176, 179, 152); (13–16) min, m/z (202, 203, 200, 101); (16–20) min, m/z (228, 226, 229, 227, 252); (20–23) min, m/z (253, 252, 250, 126); (23–28) min, m/z (276, 278, 277, 138). PAH standards and samples were analyzed in selective ion monitoring (SIM) mode for quantitative determination of analytes: Nap, m/z (128, 129, 127, 102); Acp, m/z (152, 153, 151); Ace, m/z (153, 154, 152); Flu, m/z (166, 165, 167); Phe, m/z (178, 176, 179); Ant, m/z (178, 179, 176); Flt, m/z (202, 203, 200, 101); Pyr, m/z (202, 200, 203, 101); Cry, m/z (228, 226, 229); BaA, m/z (228, 226, 227, 229, 252); BbF, m/z (252, 253, 250); BkF, m/z (252, 250, 126); BaP, m/z (252, 253, 250, 126); InP, m/z, (276, 277, 138); DBA, m/z (278, 276); and BghiP, m/z (276, 277, 138).

### Evaluating BaP Degradation Using ELISA

PMC10 was inoculated into LB broth and incubated at 37°C overnight. The overnight grown culture was centrifuged at 4,000 *g* for 10 min, and the pellet was re-suspended in 3 ml BH medium. Then 1,000 μl of the bacterial culture was added to 29 ml BH medium containing BaP at 100 ng/ml. After 20 days of incubation at 37°C in a shaking incubator, the concentration of BaP was determined using an ELISA kit (Benzo(a)Pyrene ELISA, Creative Diagnostics, USA) following the manufacturer’s protocol. Briefly, 50 μl of the standard solution or sample was added to the wells of test strips. Then, 50 μl of the enzyme conjugate solution was added to individual wells followed by the addition of 50 μl of antibody solution. Following a brief circular motion, strips were incubated at 4-8°C for 60 min. After incubation, strips were washed three times using 1X washing buffer solution. Strips were then incubated at room temperature for 30 min following the addition of 100 μl of substrate/color solution to each well. After incubation, 50 μl of stop solution was added to each well, and absorbance was measured at 450 nm using a microplate ELISA photometer within 15 min.

### Checking Clear Zones Formed by PAH Degrader

BaP degradation test was performed by observing the formation of a clear zone around isolated colonies from a BaP-containing broth culture on a BaP-coated plate. Briefly, 200 μl of 10 mg/ml BaP in acetone was spread onto Bushnell Haas minimal agar plate supplemented with trace metal and vitamins. A well was made with or without a BaP-containing BH agar. Then, 50 μl of full-grown bacterial culture was inoculated into the well and incubated at 37°C for 20 days. Degradation of BaP was recognized by clear zone formation.

### In Vitro PAH Binding Assays

PAH binding assays were performed following a previous report [1]. Briefly, 2 ml of full-grown bacterial culture (~10^9^ CFU/ml) in LB was centrifuged at 4,000 *g* for 15 min and washed with phosphate-buffered saline (PBS) twice. Finally, the bacterial pellet was suspended in 2 ml of PBS containing 10 μg/ml PAHs. Following shaking, the solution was kept at 37°C for 10 h in a shaking incubator. The PBS was maintained at pH 5.

In this study, PBS plus PAH solution was used as a positive control. Following incubation, bacteria were separated by centrifugation. The amount of unbound PAHs in the cell-free supernatant was then measured using GCMS. In the case of dead bacteria, bacterial culture (~10^9^ CFU/ml) was autoclaved at 121°C for 15 min and then centrifuged at 4,000 *g* for 15 min. After PBS washing, the experiment was done following the same protocol used for live bacteria.

### Total RNA Extraction and qRT-PCR

First, 30 mL of an overnight grown culture of *B. velezensis* was treated with 20 μg/ml BaP and incubated for 0, 24, and 48 h at 37°C. Following incubation, total RNA was extracted using an RNeasy Mini Kit (Qiagen) following the manufacturer’s instructions. Next, cDNA was prepared using an iScriptTMcDNA Synthesis Kit (Bio-Rad) and stored at -20°C for further study.

To investigate the relative gene expression levels of PAH ring-hydroxylating dioxygenase alpha subunit (RHDα) and 4-hydroxybenzoate 3-monooxygenase in response to BaP, a set of primers was used (Table 1). These primers were obtained from a previous report [17]. Relative levels of genes were determined by qRT-PCR, which was performed in a 20 μl reaction mixture. This reaction mixture contained 4 μl nuclease-free water, 10 μl SYBR Green Supermix (Bio-Rad), 5 μl cDNA, and 0.5 μl of each primer (10 μM). PCR was conducted with the following temperature profiles: activation at 95°C for 10 s, followed by 50 cycles of 95°C for 10 s, 58°C for 45 s, and elongation at 72°C for 30 s. Endogenous control gene 16S was used to normalize gene expression levels.

### Preparation of Cell-Free Culture Supernatant (CFS) and Intracellular Cell-Free Extracts (CFE)

CFS and CFE were prepared as described previously [22] with a slight modification. Briefly, cultures of probiotic strains (~10^9^ CFU/ml) were centrifuged at 10,000 *g* for 10 min. Following centrifugation, the supernatant was filtered using a 0.22 μm pore size filter. The filtered supernatant was used for further study. To prepare intracellular cell-free extracts, bacterial cells were washed twice with deionized water. Following washing, cells were suspended in deionized water. These cells were then subjected to bead beating for 1 min to break cell walls. Following centrifugation at 7,800 *g* for 10 min, cell debris was removed and the supernatant was used as an intracellular cell-free extract for further experiment.

### Cells and Culture Conditions

Mouse macrophage RAW 264.7 cells were purchased from the American Type Culture Collection (ATCC, USA) and cultured in Dulbecco’s Modified Eagle’s Medium (DMEM) supplemented with 10% (v/v) heat-inactivated fetal bovine serum (FBS) and 1% (v/v) antibiotic/ antimycotic cocktail at 37°C with 5% CO_2_. Cells were seeded into 96-well plates at a density of ~1 × 10^4^ cells/well and incubated at 37°C with 5% CO_2_ for 24 h. Human dermal fibroblast cells were purchased from the American Type Culture Collection (ATCC, USA) and maintained in Fibroblast Basal Medium (FBM) (Lonza; CC-3131) supplemented with FGMTM-2 SingleQuotsTM supplements (CC-4126) which included 0.50 ml insulin, 0.50 ml hFGF-B (human fibroblastic growth factor), 0.50 ml GA-1000 (Gentamicin sulfate-Amphotericin), and 10.00 ml FBS (heat-inactivated fetal bovine serum). Cells were maintained in a humidified atmosphere at 37°C with 5% CO_2_, seeded into 96-well plates at a density of ~1 × 10^5^ cells/well, and cultured for 24 h.

### Cell Viability Assay

Cell viability assay was performed to determine the cytotoxic effects of probiotic bacteria on RAW 264.7 macrophages and human dermal fibroblast cells. This assay was based on the conversion of a colorless, transparent 3-(4, 5-dimethylthiazol-2-yl)-2, 5-diphenyltetrazolium bromide (MTT) salt into a purple formazan crystal by mitochondrial dehydrogenase of live cells. Following 24 h of incubation, RAW 264.7 and HDF cell monolayers were seeded into 96-well plates, treated with probiotic bacteria at 1~0^5^ or ~10^7^ CFU/ml, and incubated at 37°C with 5% CO_2_ for another 24 h. Following incubation, 50 μl of 2 mg/ml MTT solution was added to each well and incubated for a further 4 h at 37°C. After incubation, media were removed completely from the plate and 100 μl of DMSO was added to each well. Plates were then incubated at 37°C for 10 to 15 min to dissolve the formazan crystal. Absorbance was measured at 570 nm using a Victor ×3 Multilabel Reader (Perkin Elmer 2030, USA) following 5 min of incubation in a shaker at room temperature (RT).

### Determination of Anti-Inflammatory Activity of Probiotic Bacteria in RAW 264.7 Macrophages

Cells were seeded into 96-well plates at a density of ~1 × 10^4^ cells/well and incubated at 37°C with 5% CO_2_ for 24 h. Following incubation, media were removed from the plate and the cell monolayer was treated with the selected probiotic strain at ~10^5^ or ~10^7^ CFU/well in DMEM without antibiotics for 12 h. As for the case cell-free supernatant (CFS) of the probiotic bacteria, the RAW 264.7 cell monolayer was treated with 10% or 20% of CFS in DMEM for 12 h. Following incubation, RAW 264.7 cells plus probiotics were exposed to 2,000 ng/ml lipopolysaccharide (LPS) for 24 h. After incubation, the production of nitric oxide (NO) was measured using Griess reagent (Sigma Aldrich, USA) following the manufacturer’s instructions. Briefly, 50 μl of the culture supernatant was transferred to a new plate. Then, 50 μl of Griess reagent was added. After 1 min of mixing in a shaker, the plate was incubated at 37°C for 15 min. Absorbance was measured at 540 nm using a Victor ×3 Multilabel Reader (Perkin Elmer 2030).

### Measuring DPPH-Free Radical Scavenging Activity

In this experiment, probiotic cell-free supernatant and cell-free extract were used as assay samples. The DPPH-free radical scavenging activity of each sample was measured by following the procedure described earlier [22]. Briefly, 0.8 ml of each sample was added with 2.2 ml DPPH-free radical (0.1 mM), mixed vigorously, and then kept in the dark for 30 min at RT. Finally, the absorbance was measured at 517 nm. Deionized water was used as a blank control sample. The DPPH-free radical scavenging activity was calculated using the following equation:

Scavenging effect (%) = [1 - A517 (sample) / A517 (blank)] × 100.

### Statistical Analysis

Every experiment was repeated at least three times. Student’s t-test was used to evaluate the statistical significance of results of different experiments using GraphPad Prism 7 software. In the graph, data are presented as mean ± SD. Means were considered to be significant at *p* < 0.05 and marked with asterisks (*p < 0.05; **p < 0.01; ***p < 0.001; and ****p < 0.0001).

## Results

### Identification of PAH-Degrading Bacteria Based on 16S rRNA Gene Sequence Analysis

From an initial screening with bacteria isolated from 26 different fermented foods, five bacterial strains (PMC1, PMC2, PMC3, PMC10, and PMC12) showed PAH-degrading efficacy (data not shown here). Molecular assays were conducted to identify these five strains. To establish the identity and classification of newly isolated bacteria, 16S rRNA gene sequences are common genetic markers frequently used in phylogeny and taxonomy [23, 24]. They are also widely used to study the taxonomic relationship between different microorganisms. Sequence similarity of 98.65% is considered as the cut-off value for delineating species [20, 23]. Thus, 16S rRNA gene sequence of PMC10, a PAH-degrading isolate, was compared with the reference sequence database from the National Center for Biotechnology Information (NCBI). PMC10 was identified as *Bacillus velezensis* (Table 2). This isolate shared more than 99% sequence similarity with its various closest relative. It showed maximum similarity (99.93%) with *Bacillus velezensis* CR-502. However, 16S rRNA gene sequence information is not always adequate to confirm bacterial identity at the genus level. Therefore, we conducted an extensive whole-genome sequence analysis to identify the exact species of the selected strain.

### Revealing the Identity of PMC10 by Comparative Genomic Analysis

**Fig. 1:**
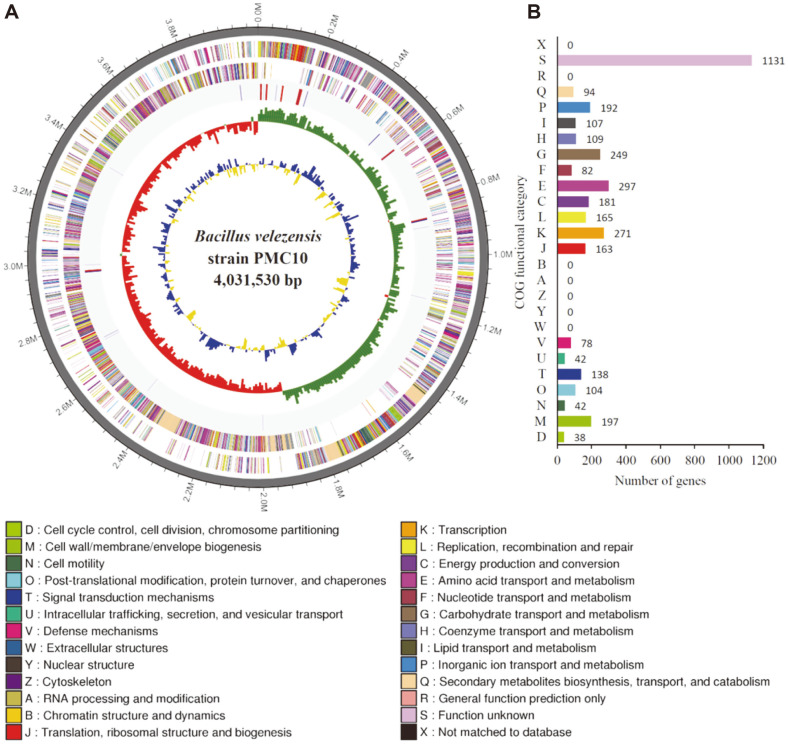
Identification of PMC10 through high-throughput whole genome sequencing. (**A**) Circular map of *Bacillus velezensis* strain (PMC10) genome. Antisense and sense strands are colored according to COG categories and placed in the outermost ring. RNA genes are shown next to antisense and sense strands from the outside where tRNA and rRNA are shown as red and blue, respectively. Inner circles present GC skew. Yellow indicates positive values and blue indicates negative values. GC content is showed in red and green. CLC genomics was used to visualize the genome map. (**B**) Relative number of clusters of orthologous groups (COG) based on functional gene categories.

Genomic analysis revealed that PMC10 possessed a single, circular chromosome of 4,031,530 bp, with an average GC content of 46.1% (Fig. 1). The PMC10 genome had around 4,077 coding sequences (CDSs) with an average length of 873.4 bp (Fig. 1A). Coding sequences were grouped into different Orthologous Group (COG) clusters based on functional prediction (Fig. 1B). A total of 3,680 proteins were assigned to COG families among these CDSs [25]. Among these proteins, biological functions were predicted for 2,549 or 62.5% of identified proteins. A total of 1131 CDS (27.7%) were found to be homologous to conserved proteins in other organisms with unknown functions. In addition, 397 (9.7%) of identified CDSs did not match with any proteins in the database with known functions. Therefore, they were defined as hypothetical proteins. Genomic analysis also predicted about 78 tRNA and 24 rRNA genes.

**Fig. 2:**
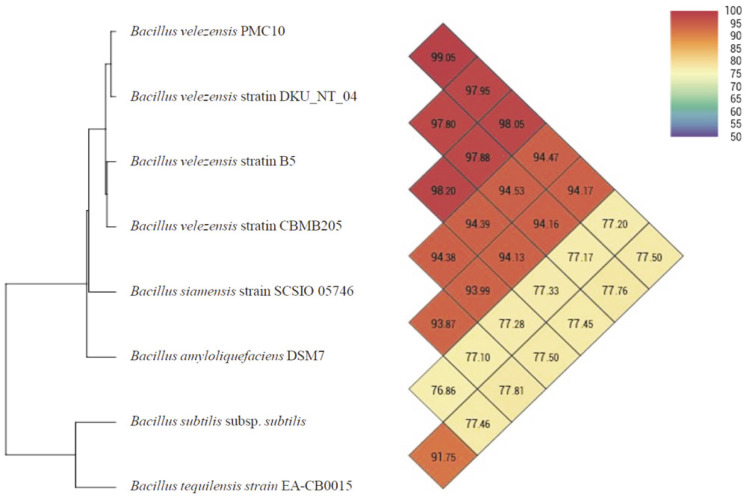
Phylogenomic tree of the selected isolate based on available genomes of *Bacillus* species. Calculated values are used to determine species. If the value is greater than 96%, it means that the strain belongs to the same species. The connecting point of diagonals departing from each strain shows the calculated OrthoANI value between two strains.

For robust and faster taxonomic identification, OrthoANI is a widely used bioinformatics tool for calculating average nucleotide identify [26]. Whole genome sequence data of PMC10 were used to identify shared similarities between strains using OrthoANI (Fig. 2). Results of OrthoANI analysis revealed that the isolated strain PMC10 shared 99.05% similarities with the genome of B. valenzesis strains (DKU_NT_04), 97.95% similarity with *B. velezensis* (strain B5), and 98.05% similarity with *B. velezensis* (CBMB205). The similarity percentage was significantly higher than the cut-off value of 95% for species delineation [27]. On the other hand, isolate PMC10 shared very lower similarities (less than 77.50%) with other *Bacillus tequilensis* genomes. These results of genomic analyses suggest that this PAH-degrading isolate PMC10 is *B. valenzensis*, similar to the 16S rRNA gene sequencing result.

Genome features of PMC10 (*B. velezensis*) were then compared with other publicly available genomic information of different *B. velezensis* strains (9D-6, UFLA258, NST6 and AL7) (Table 3). Although all strains belonged to the same species, their genome sizes, GC contents, and numbers of CDS, rRNA, and tRNA, were very different. These data suggest that PMC10 is a new strain of *B. velezensis*. The draft genome sequence of strain PMC10 will further guide us to unveil the molecular mechanism involved in PAH degradation by PMC10.

### Degradation of BaP in Broth Culture and Agar Medium

**Fig. 3:**
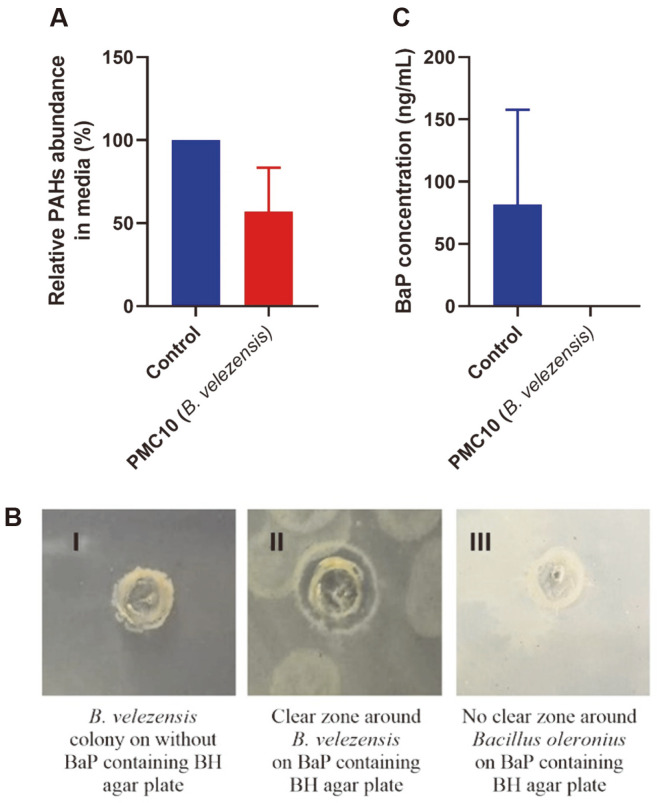
Biodegradation of benzo(a)pyrene by *B. velezensis*. (**A**) GCMS data representing relative BaP abundance in BH media following co-incubation with *B. velezensis* for 20 days. (**B** I) *B. velezensis* strain was inoculated onto BH agar plate without BaP, showing no clear zone. (**B** II) *B. velezensis* formed a clear zone of degradation around the colony on BaPcontaining BH agar plate. (**B** III) No clear zone of degradation was formed around other bacteria tested on BaP-containing BH agar plates. (**C**) ELISA data showing BaP abundance in BH media following co-incubation with *B. velezensis* for 20 days.

The BaP degradation abilities of isolates PMC1, PMC2, PMC3, PMC10, and PMC12 were evaluated using 50 ml BH broth containing 10 μg/ml BaP as the sole source of carbon. Following 20 days of incubation with or without a test strain, the concentration of the polyaromatic hydrocarbon in the broth culture was measured by GCMS analysis. Changes in BaP concentration in the test cultures were compared with control experiments lacking the probiotic strain. According to GCMS data, PMC10 resulted in the highest reduction in the concentration of BaP after 20 days of incubation among all strains tested. It reduced 51.32% of the dissolved BaP compared to the control group (Fig. 3A).

To confirm the effect of PMC10 as a BaP degrader, we inoculated the probiotic strain into a well of BaP-coated BH minimal agar plate. Following 20 days of incubation, PMC10 showed a clear zone of degradation of BaP around the bacterial colony (Fig. 3B-II). Meanwhile, it did not result in a similar pattern on BH agar without BaP coating. These results further prove the ability of PMC10 to degrade polyaromatic hydrocarbons such as BaP.

### PMC10 Mediated BaP Degradation in a Broth Culture Measured by ELISA

To re-evaluate the BaP degradation efficiency of PMC10, PMC10 was co-incubated with 100 ng/ml BaP for 20 days. Following incubation, the amount of BaP was measured using ELISA in control or PMC10-treated sample. In comparison with the control group, PMC10 completely degraded BaP in the BH broth medium (Fig. 3C).

### Ability of PAH to Remove Live and Dead PMC10

**Fig. 4:**
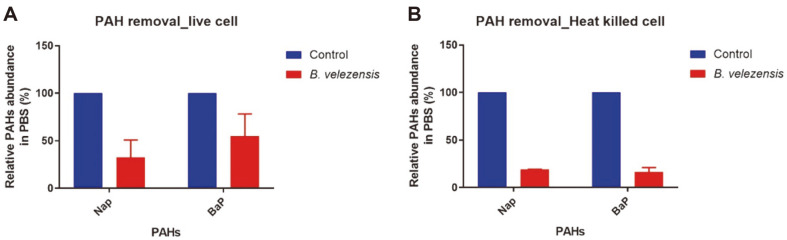
Detoxification of benzo(a)pyrene and naphthalene by *B. velezensis*. (**A**) GCMS data showing relative abundance of Nap and BaP following live *B. velezensis* mediated removal in PBS. (**B**) GCMS data showing relative abundance of Nap and BaP following heat-killed *B.subtilis* mediated removal in PBS.

To check the ability of PMC10 to remove PAHs, two PAHs, namely naphthalene (Nap) and benzo(a)pyrene (BaP), were selected. Live bacterial cells were mixed with each PAH at 10 μg/ml in PBS and the concentration of the PAH after removal was measured by GCMS analysis. Results showed that live PMC10 bacteria significantly reduced the amount of PAH in comparison with the control. Live PMC10 reduced 67.60% and 45.04% of the amount of dissolved Nap and BaP, respectively, by binding to PAH in comparison with the control group (Fig. 4A). Heat-killed PMC10 also significantly reduced aqueous PAH in comparison with the control group. Heat-killed PMC10 reduced 81.00% and 83.45% of the amount of dissolved Nap and BaP, respectively, by binding to PAH in comparison with the control group (Fig. 4B).

### Relative Gene Expression Profile in Response to BaP Exposure

To gain insight into the possible mechanism involved in BaP degradation by PMC10, relative expression levels of RHDα and HMBO genes were investigated using real-time quantitative PCR.

**Fig. 5:**
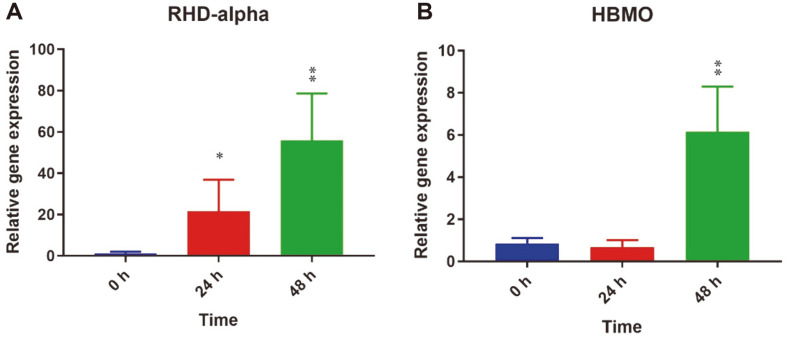
Relative gene expression in *B. velezensis* in response to BaP. *B. velezensis* cells were co-incubated with BaP. Induction of (**A**) RHDα, and (**B**) HBMO gene expression was studied by quantitative real-time PCR. Data are presented as mean ± SD of three independent experiments done in triplicate. *p < 0.05; **p < 0.01 by Student’s *t*-test.

In this experiment, an overgrown culture of PMC10 was treated with 20 μg/ml BaP for a different time period. Data clearly showed that the expression of gene RHDα was upregulated in response to BaP treatment. In comparison with the control group (0 h), PMC10 increased the gene expression level of RHDα by 20.39-fold (*p* < 0.05) at 24 h after treatment. At 48 h after incubation, PMC10 increased RHDα gene expression by about 54.59-fold (*p* < 0.01) (Fig. 5A). HBMO gene expression was also increased in response to BaP treatment. After 24 h of incubation, the expression of HBMO gene was not increased. However, after 48 h of incubation, gene expression was increased significantly by 5.31-fold (*p* < 0.01) in comparison with the control group at 0 h (Fig. 5B).

### Cell Cytotoxicity of PMC10 to RAW 264.7 Macrophages and HDF Cells

**Fig. 6:**
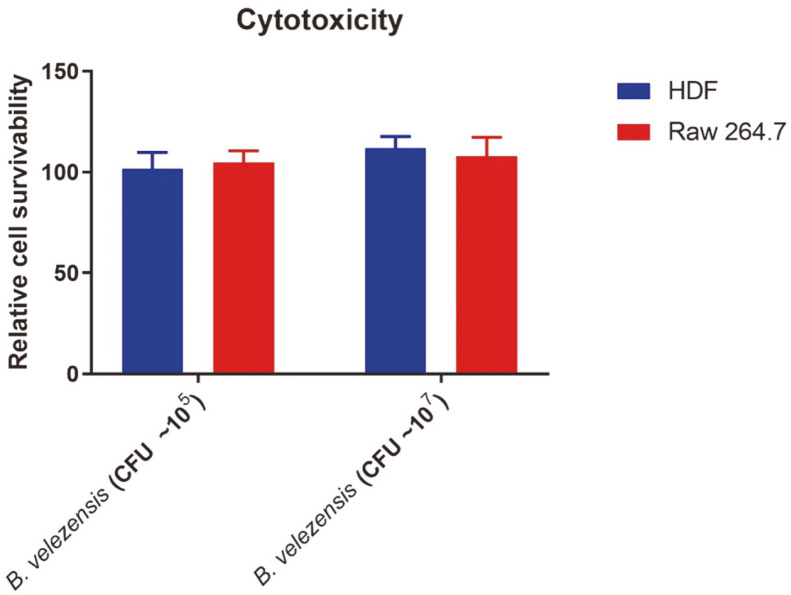
Assay for cell cytotoxicity in human dendritic fibroblast (HDF) and RAW 264.7 cells. HDF and RAW 264.7 cell monolayers were treated with live *B. velezensis*. Relative viabilities of HDF and RAW 264.7 cells were measured using MTT assay.

The cytotoxic effects of PMC10 at 10^5^ and 10^7^ CFU/ml on Raw 264.7 and HDF cells were examined using MTT assay. Results showed that PMC10 at 107 CFU/ml was not cytotoxic to Raw 264.7 macrophages or HDF cells (Fig. 6).

### Anti-Inflammatory Activities of PMC10 Strains

**Fig. 7:**
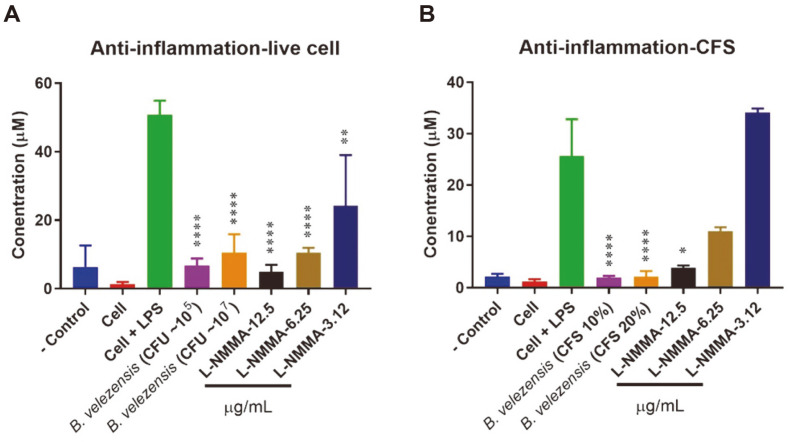
Modulation of LPS-induced NO production by live *B. velezensis* and cell-free supernatant of *B. velezensis* in LPS-treated macrophages. RAW 264.7 macrophages were treated with LPS at a concentration of 2,000 ng/ ml. The production of NO was measured following treatment with (**A**) live *B. velezensis* or (**B**) cell-free supernatant of *B. velezensis*. Data are presented as mean ± SD of three independent experiments done in triplicate. *p < 0.05; **p < 0.01; ***p < 0.001; and ****p < 0.0001 by Student’s *t*-test. L-NG-monomethyl arginine acetate (L-NMMA) was used as a positive control.

Nitric oxide (NO) is a pro-inflammatory mediator that can induce inflammation [28]. Therefore, in this experiment, the effects of live cell and cell-free supernatant of PMC10 on the production of NO in LPS-stimulated macrophages were investigated. In the case of live cells, ~10^5^ and ~10^7^ CFU/ml of PMC10 isolate significantly (*p* < 0.0001) inhibited the production of NO. In this experiment, L-NMMA, a potent NO synthesis inhibitor, was used as a positive control. It also reduced the production of NO at a concentration up to 3.12 μg/ml (*p* < 0.01) (Fig. 7A). In the case of cell-free supernatant, 10% and 20% CFS of PMC10 isolate significantly (*p* < 0.0001) inhibited the production of NO in LPS treated RAW 264.7 cells. Under this assay condition, 12.5 μg/ml L-NMMA also reduced the production of NO (*p* < 0.05) (Fig. 7B).

### Antioxidative Activity of PMC10

**Fig. 8:**
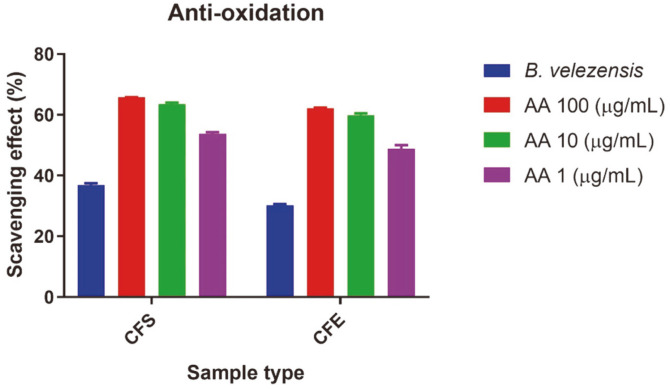
Free radical scavenging effects of cell-free supernatant (CFS) and cell-free extract (CFE) of *B. velezensis*. DPPH-free radical scavenging effect of ascorbic acid (AA) as a positive control was also measured.

The antioxidative potential of PMC10 isolate was evaluated by performing DPPH-free radical scavenging assay. Cell-free supernatant of PMC10 isolate showed a potential free radical scavenging effect of 36.9% in comparison with the control group using DW instead of CFS. In this experiment, ascorbic acid (AA) was used as a positive control. AA at 100, 10, and 1 μg/ml showed scavenging effects of 65.74%, 63.47%, and 53.79%, respectively (Fig. 8). The cell-free extract of PMC10 isolate also exhibited potential DPPH free radical scavenging effect of 30.26% in comparison with the control group. Here, 100, 10, and 1 μg/ml of AA as a positive control showed scavenging effects of 62.18%, 59.90%, and 48.85%, respectively (Fig. 8).

## Discussion

Polycyclic aromatic hydrocarbons (PAHs) such as naphthalene, benzo(a)pyrene, pyrene, fluorine, and so on are omnipresent environmental pollutants that are generally produced by inadequate combustion of organic materials such as coal, wood, oil, and petrol [9]. Other major sources of PAHs include motor vehicle exhaust, residential heating, coke and aluminum production industry, and petroleum refineries [9].

PAHs are highly toxigenic, carcinogenic, and mutagenic in nature [29]. Long-term exposure to gaseous or particulate PAH mixtures in the air can lead to severe health problems such as skin cancer [5], digestive tract cancer [30], cardiovascular disease, fatal ischemic heart disease, heart rate variability, hypertension, inflammation, atherosclerosis, and asthma [31].

Therefore, finding a safe and effective option to degrade and/or remove PAHs from the human body is urgently needed. Recently, scientists are very interested in xenobiotic-degrading microorganisms for bioremediation and removal of toxic chemicals [32].

To this end, we have chosen probiotic bacteria isolated from different Korean fermented foods as ideal candidates to degrade and/or remove PAHs. By definition, probiotics are live bacteria that confer a significant health benefit to consumers or hosts [33]. Probiotics are used for the improvement of intestinal health, immune response, reduction of serum cholesterol, prevention of cancer, treatment of acute or antibiotic-associated diarrhea, improvement of lactose metabolism, and so on [10]. In addition, probiotic bacteria are used for detoxifying xenobiotics and adsorbing toxic molecules by physical interactions [1].

In this study, we selected benzo(a)pyrene (BaP) as a representative PAH compound because BaP is a carcinogenic compound that has been comprehensively studied and classified by the IARC as a Group 1 carcinogen [5]. We also selected naphthalene, another common pollutant, because it has been frequently used as a chemical model to study the degradation of PAHs [16].

To find a prospective probiotic candidate with potential BaP-degrading efficacy, we chose a total of 26 fermented foods and co-incubated them with BaP at 250 mg/l for 5 times of 7-day successive incubation in Bushnell Haas minimal broth. Following incubation, bacterial culture was spread onto NA agar plates, and colonies with distinct morphologies were selected for further studies. Since the BH media contained BaP as the sole carbon source, live bacteria in this condition could have the ability to degrade BaP to get essential energy. Initially, we isolated five distinct colonies: PMC1, PMC2, PMC3, PMC10, and PMC12. Based on 16S rRNA gene sequencing, PMC10 was identified as *Bacillus velezensis*. Comparative whole genome sequencing analysis also revealed the identity of PMC10 as *B. velezensis*. All bacterial isolates were co-incubated with BaP separately in BH medium to check their BaP-degrading efficacies. GCMS analysis data revealed that isolate PMC10 showed the highest potential BaP degradation efficacy (51.32%) in comparison with the control group excluding bacteria. To validate this GCMS analysis result, the strain PMC10 was inoculated on a BaP-coated BH agar plate to observe its degradation efficacy on agar condition. In the BaP-containing agar plate, PMC10 isolate degraded BaP around the bacterial colony and formed a clear zone. Degradation of BaP by PMC10 was also measured by ELISA. Results showed complete degradation of BaP (100 ng/ml) in comparison with the control group. These results suggest that PMC10 is a promising probiotic candidate with potential PAH degradation efficacy. In a previous study, *Paracoccus yeei*, *Acinetobacter lwoffii*, *Micrococcus luteus*, *Staphylococcus caprae*, *Pseudomonas oleovorans*, and *Bacillus licheniformis* have shown potential BaP-degrading efficacy [6]. *Pseudomonas aeruginosa* also shows degradation efficacy against Nap [16]. LAB have also been reported as potential probiotics to degrade different PAH, including BaP and Nap [12].

It has been reported that PAH ring-hydroxylating dioxygenases (PAH-RHDalpha) and 4-hydroxybenzoate 3-monooxygenase (HBMO) are involved in the initial step of aerobic metabolism of PAH [17, 34]. To gain insight into the mechanism of BaP degradation by PMC10, we performed gene expression profiling in response to BaP using two sets of primers, RHDα and HBMO. Strain PMC10 clearly induced RHDα gene expression after incubating with BaP for 24 h and 48 h. HBMO gene was also induced following 48 h of incubation. In another study, RHDα and HBMO gene expression levels were found to be increased in *Novosphingobium pentaromativorans* US6-1 in the presence of BaP [17]. These results suggest that the degradation of BaP can be accomplished by ring-hydroxylating dioxygenase-initiated metabolic pathway. A previous study also suggested that this pathway enters the tricarboxylic acid (TCA) cycle [17].

In addition to biodegradation, we were also interested in the removal of PAHs such BaP and Nap by isolate PMC10. We co-incubated bacteria with BaP and Nap. Following centrifugation, the removal effect was compared with the control group excluding bacteria. Bacterial number and pH of the media were selected based on a previous study [1]. GCMS analysis results suggested that live PMC10 was capable of removing BaP (45.05%reduction) and Nap (67.61% reduction) significantly. Heat-killed PMC10 was also capable of removing BaP (83.45% reduction) and Nap (81.00% reduction) in comparison with the control group. These data suggest that PMC10 is a prospective candidate to detoxify food or skin PAH contamination. A previous study has shown that lactic acid bacteria such as *Bifidobacterium lactis*, *Lactobacillus acidophilus*, *Lactobacillus bulgaricus*, and *Streptococcus thermophilus* have potential BaP removal efficacy [1]. Other studies have also examined the potential of probiotic bacteria to remove food-induced toxicants, mycotoxins, and heavy metals [35-37].

Although PMC10 was isolated from fermented food, to evaluate its safety further, we checked its toxicity to RAW 264.7 macrophages and human dermal fibroblasts. Cell viability data suggested that live bacteria of strain PMC10 at ~10^5^ and ~10^7^ CFU/ml were not cytotoxic to RAW 264.7 macrophages or HDF cells. As PAHs are potential skin carcinogenic agents [5], we therefore tested dermal fibroblast cells to determine the possible scope in utilizing the strain PMC10 to degrade skin PAHs.

In addition, various functional properties such as anti-inflammation and anti-oxidation effect of the selected strain were investigated. Nitric oxide is a crucial marker in inflammatory disease. Therefore, the effects of PMC10 live cells and cell-free supernatant on NO production in LPS-treated RAW 264.7 macrophages were studied. Both live bacteria and cell-free supernatant of PMC10 showed significant anti-inflammatory effects by lowering NO production. L-NG-monomethyl arginine acetate (L-NMMA), a nitric oxide synthase inhibitor [38], was used in this study as a positive control. Interestingly, PMC10 showed better efficacy than 6.25 μg/ml L-NMMA. A previous study also reported that different probiotics such as *Lactobacillus acidophilus*, *Lactobacillus casei*, *Lactococcus lactis*, *Lactobacillus reuteri*, *L. rhamnosus* GG, and *Saccharomyces boulardii* have potential anti-inflammatory activities [39].

In addition, cell-free supernatant and cell-free extract of PMC10 showed potential radical scavenging activities. Ascorbic acid, a well-known free radical scavenger [40], was used as a positive control in this study. The antioxidative effect of PMC10 was comparable to that of ascorbic acid. Some probiotics have the ability to reduce oxidative damage by scavenging free radicals or by modulating activities of crucial antioxidative enzymes. Thus, cells can be protected from carcinogen-induced damage [41]. *Bifidobacterium longum, Lactobacillus acidophilus*, and *Lactobacillus rhamnosus* have been reported to possess potential antioxidative effects [42].

Altogether, our results suggest that *B. velezensis* is safe, and our study also highlights the potential application of *B. velezensis* as a probiotic to degrade or detoxify PAHs in the human gut or skin. Further studies are required to purify responsible enzymes, which can be formulated as cosmetics in the future to detoxify PAH on human skin. Besides, our study showed that along with the PAH degradation efficacy, *B. velezensis* has anti-oxidation and anti-inflammatory potential, which will benefit human cells during application.

## Figures and Tables

**Table 1:** List of primers used in this study for qRT-PCR.

Primer	Sequence 5'-3'	Amplicon size (bp)
RHDα Fw	GGA AAG GCT TGT GGG TGT CG	252
RHDα Re	GTG GCA TCA TCG CAT CGT GT	
HBMO Fw	GCG TGT GCC GCC TTG TAA TCA	157
HBMO Re	ACG CCA GTT CGT CCC AGA TGC	
16S Fw	AAC GCG AAG AAC CTT AC	433
16S Re	CGG TGT GTA CAA GAC CC	

Fw, Forward primer; Re, Reverse primer; RHDα, ring-hydroxylating dioxygenase alpha subunit; HBMO, 4-hydroxybenzoate 3- monooxygenase.

**Table 2:** Identification of isolated bacterial strain based on 16S rRNA gene sequence analysis.

Rank	Name	Strain	Pairwise similarity (%)	Different nt/ Total nt	Completeness (%)
1	*Bacillus velezensis*	CR-502	99.92722	1/1374	95.38043
2	*Bacillus velezensis* subsp. *subtilis*	KCTC 13613	99.78571	3/1400	100
3	*Bacillus siamensis*	CR-502	99.78571	3/1400	100
4	*Bacillus amyloliquefaciens*	DSM 7	99.64286	5/1400	100
5	*Bacillus tequilensis*	KCTC 13622	99.57143	6/1400	100

**Table 3:** Comparison of genome features of *Bacillus velezensis* strains.

Strains	PMC10	9D-6	UFLA258	NST6	AL7
Genome size (bp)	4,031,530	3,960,000	3,947,620	4,141,240	3,894,709
G+C content (%)	46.1	46.5	46.5	46.04	46.64
Predicted CDS	4,077	3849	3,747	4070	3706
Number of rRNA genes	24	21	116	27	27
Number of tRNA genes	78	68		86	86
References		[43]	[44]	[45]	[46]
